# Notes

**Published:** 1902-07

**Authors:** 


### NOTES.

### All Hail the Microbe.

Go draw the curtains, sister, and stop up all the chinks,
For microbes and bacilli are kicking up high jinks;
Go sterilize the water and disinfect the cook—
The germ is grimly stalking like some pursuing spook!

And while you're doing these things,
You'd better do 'em twice—
And when you've got 'em finished,
Go down and boil the ice!

Be careful of the mutton (Oh, guard ye well the meat!)
It's full of varied microbes we would not care to eat!
And trace the antecedents of that seductive stew—
We know not how much danger is lurking in the brew!

Go, vaccinate the oatmeal
And sulphurize the rice;
And once again, dear sister,
DON'T fail to BOIL the ICE!

Dr Rhoads, of the Springfield track, could not see why the votes on dates for racing were not cast according to his views. Well, he talked too much and said less.—*Quaker Quality.*

State Veterinary Surgeon Knowles, of Montana, takes a keen interest in the National Live Stock Association, and his urging maintenance of the ninety-day quarantine by our government on all importations of stock for the protection of our great cattle interests met with success and gained for him the hearty approval of the owners of Montana's herds.

*The Cow-Pea* is the title of the latest publication issued by the Experiment Farm of the North Carolina State Horticultural Society at Southern Pines, N. C. This book is plainly written, and intelligently discusses the value and uses of this important crop, the cow-pea. Our readers can secure a copy free by writing to the Superintendent of the Experiment Farm, Southern Pines, N. C.

Civil service examinations will be held in the usual cities on October 7th for meat-inspectors for the meat-inspection service of the Bureau of Animal Industry.

The horse is doomed. Mr. Edison is confident he will have every one of the species out of the business as soon as he gets his battery for motor vehicles in the market. This untimely end of the horse has long been prophesied, but he is still as much in demand to-day as at any time since he was trained to the offices of men. I think it will be many and many a day before we witness the passing of the equine. There are, and doubtless always will be, a large number of people who have a preference for the horse over electric or gasoline motors, and it is not wise to predict that man's best friend is shortly to be cast aside. Besides, why put away the horse? There is room enough and plenty of work for him to do. Man's ingratitude would surely not be so base as to lead him to turn his back upon so faithful a friend.—*Quaker Quality.*

*Hydrophobia Scares.* There is no special hydrophobia season. In the hot months dogs suffer from intense heat and are liable to heat stroke, especially if they be unable to obtain water. No doubt the so-called rabid dogs are aften the victims of other disorders; but the germs of rabies are no more active and general in summer than in winter. Although this fact has been presented to the public repeatedly, every dog which is plainly not in good health in July and August is pursued to its death by police and public.

There are specialists in diseases of the nervous system who are disposed to doubt whether there is such a disease as hydrophobia, because of the number of reported cases which they have found to be due to some other malady or to fright. Anyhow, it may be said with emphasis that cases of true rabies in dogs and hydrophobia in man are extremely rare, and that a considerable majority of alleged hydrophobia deaths might be traced to panic. In the instances where the patients actually bark, crawl on all fours, and imitate the dog, it is safe to say there is no hydrophobia. In their fear of the malady such patients exhibit all the symptoms which they believe are displayed by the hydrophobia patient. The latter suffers from a nervous constriction of the throat which is increased by any attempt to swallow. This is the source of the alleged fear of water and of the name of the disease. This constriction of the larynx produces the noise which is popularly likened to the barking and growling of a dog. It also causes a struggle for breath, which is assumed to be an effort to bite the persons in attendance.

It is almost certain that the popular fallacies about mad dogs and hydrophobia have killed more persons than has hydrophobia itself. Fairly sensible persons when bitten by a cross dog often become panic stricken and die in great agony after exhibiting many of the alleged symptoms of hydrophobia, although the dog in each case may have been free of rabies. A typical instance was that of a Chicago woman whose malady was diagnosed by two doctors as hydrophobia. After her death the dog which bit her was hunted up and was found to be entirely well. Intelligent people in every community should stoutly combat every mad dog scare. Instead of having an alleged mad dog shot, they should shut him up until it shall be determined whether he has rabies. This would often secure persons who may have been bitten against death from fright. —*Philadelphia Record*, July 3, 1902.

